# 

**Published:** 1918-12-14

**Authors:** 


**ORDER THE PAPER.**

**In consequence of the No Returns Order of the Government, readers of THE HOSPITAL are requested to ensure a regular supply of the paper by placing an order with their newsagent or bookstall without fail.**

